# Editorial for the Special Issue on Advanced Thin-Films: Design, Fabrication and Applications

**DOI:** 10.3390/mi15020255

**Published:** 2024-02-09

**Authors:** Elena Kalinina

**Affiliations:** 1 Laboratory of Complex Electrophysic Investigations, Institute of Electrophysics, Ural Branch of the Russia Academy of Sciences, 620016 Yekaterinburg, Russia; jelen456@yandex.ru; Tel.: +7-343-267-87-82; 2 Department of Physical and Inorganic Chemistry, Institute of Natural Sciences and Mathematics, Ural Federal University, 620002 Yekaterinburg, Russia

The application scope of basic functional materials can be expanded through the creation of thin films due to the emergence of new unique properties of film materials that differ from their bulk analogues. Promising thin films, including composite and nanostructured ones, find application in electronic devices, microelectromechanical systems (MEMSs) and sensors and catalytic, piezoelectric, acoustic and optoelectronic devices, in the creation of actuators, as well as in systems using high-temperature superconductors. Moreover, functional film materials are used in energy generation and storage systems, including all-solid-state batteries (ASSBs), solar cells and hydrogen energy.

Thin film technologies are based on various principles such as epitaxial deposition, ion beam-assisted deposition (IBAD), electron beam evaporation, atomic layer deposition (ALD), plasma discharge sputtering, reactive DC magnetron sputtering, electrodeposition, pulsed laser ablation, chemical bath deposition, spray pyrolysis, and electrophoretic deposition (EPD).

This Special Issue, “Advanced Thin-Films: Design, Fabrication and Applications”, contains 13 original articles devoted to various aspects of the formation of advanced thin films, the determination of their functional properties and the characteristics of devices based on them. The presented articles focus on important issues related to the following areas of thin film applications: flexible electronic devices, high-temperature superconductors, gas sensors, photocatalysts, biocompatible piezoelectric generators, optical devices, protective coatings, piezoelectric film actuators, solar cells and all-solid-state batteries. The obtained and published results of these contributions are briefly summarized and presented below.

The creation of ASSBs is an attractive way to solve the safety problems of Li-ion batteries through the use of liquid or polymer electrolytes during long-term operation and expand the range of their operating temperatures [1,2,3]. Compounds based on Li_7_La_3_Zr_2_O_12_ (LLZ) are promising solid electrolytes for all-solid-state batteries due to their high values in terms of lithium-ion conductivity. Reducing ohmic losses in the solid-state electrochemical cell can be achieved through the thin film design of the electrolyte membrane. E. Lyalin et al. (Contribution 1) applied the promising EPD method to form thin films of an LLZ electrolyte on different substrates such as Ti, Ni and steel substrates. After annealing in an Ar atmosphere, differences in the phase composition of the LLZ films depending on the type of substrate were observed. It was shown that the additional non-conducting Li_2_CO_3_ phase does not appear in the LLZ films deposited on Ti substrates, and the highest values of lithium-ion conductivity were achieved for these thin films.

Nanostructured films based on ZnO exhibit photoelectric activity during the decomposition of water and can be used for hydrogen production. Photoelectrochemical methods of hydrogen generation are widely used in hydrogen and solar energy [4]. Thus, the determination of approaches and design strategies for the efficient production of solar cells at a low cost is extremely relevant. S.I. Al-Saeedi (Contribution 2) prepared ZnO nanorod-arrayed films and studied the influence of the structure and morphology of the obtained films on the efficiency of hydrogen generation. 

Due to their properties, MASnI_3_ and CsPbI_3_ perovskites are considered promising materials for solar cells. In their work, K.H. Mahmoud et al. (Contribution 3) modeled solar cells, and their charge transfer mechanisms and cell characteristics were determined, including open circuit voltage and C-V characteristics for the most efficient energy conversion. The influence of annealing on perovskite film materials, which determines their temperature instability, has been established.

Oxygen sensors are used in many industries, including metallurgy, the chemical industry, and mechanical engineering. Thin films are a promising technology in terms of the miniaturization and scale-up of O_2_ gas sensor production. The resistance of TiO_2_ films depends on the oxygen content in the gas atmosphere, so they can be used when creating sensors. Plasma-enhanced atomic layer deposition (ALD) technology is of particular interest for the formation of thin films of titanium dioxide TiO_2_ on SiO_2_/Si substrates. The TiO_2_ films obtained by A.V. Almaev et al. (Contribution 4) demonstrated high sensitivity to oxygen in the range of 0.1 to 100 vol. %. A mechanism describing the effect of oxygen concentration on film resistance has been proposed.

The creation of autonomous devices using generators based on piezoelectric materials is in great demand since such devices can operate independently of batteries and other power sources. Piezoelectric generators provide electrical energy generation through human movement and can be installed in compact health monitoring devices, sensors and other portable electronic devices [5,6]. In this regard, it is of interest to create biocompatible composite piezoelectric materials based on Na_0.5_Bi_0.5_TiO_3_ (NBTO) nanoparticles in biopolymer–chitosan composites; this study was carried out by J. Zidani et al. (Contribution 5), and the piezoelectric, optical and dielectric properties of the composite structures were determined.

Composite thin-film materials with ferroelectric properties are used as actuators to control the shape of optical reflectors. To create such shells, the use of composite structures in the form of ferroelectric polymer films poly(vinylidene fluoride-co-hexafluoropropylene) (PVDF-HFP) or poly(vinylidene fluoride-trifluoroethylene) (PVDF-TrFE) on a polyethylene terephthalate (PET) substrate was studied. Research and modeling of thin-film actuators was carried out by K. Wang et al. (Contribution 6), and the problem of creating polymer reflectors based on the proposed film structures was discussed.

In the field of creating thin-film superconducting layers of REBa_2_Cu_3_O_7-x_(REBCO), the formation effect of MgO-based surface layers on a base substrate (Hastelloy) was investigated. Devices based on high-temperature superconducting YBa_2_Cu_3_O_7-x_ (YBCO) maintain a superconducting state up to a temperature of 77 K at a current density below the critical value. High-temperature superconductors exhibit broad application prospects in the fields of powerful electromagnets, electric motors, and also power lines. F. Yu et al. used ion beam-assisted deposition (IBAD) technology as well as electron beam evaporation technology to form the textured films (Contribution 7).

In their research, D. Park et al. considered the formation of mechanical stresses in periodic structures with nanogaps on stretchable substrates; this knowledge can be used in the field of electronic and optical devices. The model used included a set of nanogaps (10 nm) on a flexible polydimethylsiloxane (PDMS) substrate coated with a 100 nm thick layer of gold. A calculation of mechanical stresses was performed based on two-dimensional finite element analysis (2D finite element analysis) (Contribution 8).

Titanium dioxide exhibits photocatalytic activity under ultraviolet light, which determines its use in the field of environmental purification [7,8,9]. T.G. Getnet et al. used the plasma spraying of TiO_2_ powder simultaneously with the plasma polymerization of hexamethyldisiloxane (HMDSO). The influence of plasma excitation energy on the properties of the obtained composite films, thickness, roughness and wettability, was studied (Contribution 9).

Hafnium oxide thin films have attracted significant attention as promising materials for optical devices due to their optical properties, chemical and mechanical stability, high hardness and laser damage resistance [10,11]. HfO_2_ thin films on quartz substrates were obtained via the magnetron sputtering of an Hf target in an oxygen atmosphere by Y. Xi et al. (Contribution 10). The effect of various negative biases on the substrate during direct current deposition was studied. It was established that setting the negative bias voltage at certain values (−25 and −50 V) leads to increased density of the HfO_2_ films.

The electrodeposition of nickel-based coatings is used in a wide range of technical applications due to their operational properties, such as resistance to corrosion, high hardness and wear resistance. Electrodeposition technology is flexible and widely used in industrial production, including mechanical engineering and electronic devices [12,13]. Interesting results on the modification of Ni were shown in the research carried out by V. Tseluikin et al. (Contribution 11). The co-electrodeposition of Ni and graphene oxide (GO) particles was carried out, and the effect of microwave treatment on the obtained Ni-GO composite coatings was investigated. An increase in the hardness of Ni-GO composite coatings was established, which is important in terms of technical applications. However, the obtained composite coating showed a decrease in corrosion resistance compared to the original Ni coating.

CdS/Si heterostructures are of significant interest in the field of optoelectronic devices [14]. CdS thin films were obtained via pulsed laser ablation in a liquid (PLAL) using a Dimethyl sulfoxide (DMSO) solution into which a Cd target was placed. F.H. Alkallas et al. studied the current–voltage and optical characteristics of the obtained structures, and the possibility of creating photodetectors based on them was confirmed (Contribution 12).

Due to their unique properties, AlN films have a very wide range of applications in electronic devices, microelectromechanical systems (MEMSs) and acoustic resonators [15,16]. The epitaxial growth of AlN films on the surface of sapphire substrates coated with molybdenum was studied by J. Kim et al. (Contribution 13). It was established that highly oriented Mo thin films provide the transfer of crystallographic information from the sapphire substrate to the AlN film during epitaxial growth. The obtained results can be used to create AlN films of high crystallinity for MEMS devices.

The topics covered in the Special Issue are presented in Figure 1.

The papers presented in this Special Issue are of interest to the scientific community and can guide future efforts to create new film structures with unique functional properties. I would like to express my gratitude to the authors, who provided insight into the problems and their proposed solutions in various applications of thin films, and I would also like to thank the reviewers and editors who helped improve the submitted papers and made significant contributions to our Special Issue in *Micromachines*.

## Figures and Tables

**Figure 1:**
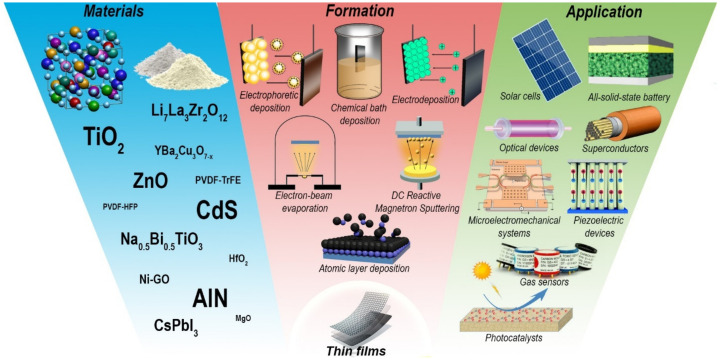
Application areas of promising thin films, methods of their formation and used materials, according to articles published in the Special Issue «Advanced Thin-Films: Design, Fabrication and Applications».
